# Extremely Rare Blood Types Resulting in Non-compatibility in the Perioperative Surgical Setting: A Case Report

**DOI:** 10.7759/cureus.79346

**Published:** 2025-02-20

**Authors:** Christopher M Russo, Edgar Villaruel, Thomas M Kane, Andrew J Evans, Patrick Coleman

**Affiliations:** 1 Anesthesiology, Walter Reed National Military Medical Center, Bethesda, USA; 2 Anesthesiology, Uniformed Services University of the Health Sciences, Bethesda, USA

**Keywords:** jr(a) antigen, junior blood group system, obstetric anesthesiology, rare blood types, transfusion therapy

## Abstract

Peripartum patients with rare blood groups (such as the Junior negative, Kell, Kidd, and Lewis antigen blood groups) can be extremely difficult to care for, even when appropriately typed and screened, crossed, and antibody tested for blood type compatibility. Here, we present a case report of a 37-year-old gravida 2 para 1 with one prior full-term singleton birth presenting for a scheduled cesarean section who was identified through routine preoperative testing to have an extremely rare blood type: Junior negative with the presence of anti-Junior antibodies. The Junior negative blood type is incompatible with over 99% of all blood products as the Junior antigen is very high frequency in almost all populations and present universally on red blood cells irrespective of ABO and rhesus (Rh) blood type. The hematologic and perioperative considerations of the patient will be discussed in this case report to include pathophysiology of the Junior-encoding gene, patient maternal hemorrhage, contingency planning, blood transfusion planning, intraoperative management, and patient outcome.

## Introduction

Rates of cesarean sections (CS) continue to increase from an estimated 7% of worldwide deliveries in 1990 to an estimated 21% in 2024 [1]. CS is therefore now more frequently performed in patients at higher risk for maternal morbidity to include parturients with advanced maternal age, obesity, and chronic medical conditions [2]. Even when indicated, CS poses several risks of complication to include uterine atony, vascular injury, uncontrolled intraoperative hemorrhage, postpartum hemorrhage (PPH), transfusion reactions secondary to blood product administration, ureteral and bladder injury, amniotic fluid embolism, hemorrhage requiring cesarean hysterectomy, and death [3]. Though the incidence of these major complications is rare, as more CS are performed on parturients with comorbidities, additional perioperative vigilance and planning will be needed. One such consideration is mitigating risk of perioperative hemorrhage. Intraoperative hemorrhage and PPH are estimated to occur in 5-10% of all CS and can range from a hemodynamically stable intraoperative patient to a catastrophic hemorrhage patient requiring arterial embolization or emergency cesarean hysterectomy. It is important to identify patients with elevated risk of hemorrhage, and it is equally important that healthcare teams are prepared to rapidly, safely, and adequately administer blood product resuscitation.

Preparing for blood product administration often begins with pretransfusion testing: a complex, multidisciplinary protocol designed to optimize perioperative safety by ensuring patients have compatible blood products readily available prior to their surgery. A nuanced challenge for both the surgeon and anesthesiologist is the perioperative care of a patient identified to have a rare blood group during pretransfusion testing. A rare blood group is one that is not typically identified on a type and screen or crossmatch. Type and screen and crossmatching results in the donor-recipient compatibility of ABO, rhesus (Rh), Kidd, Kell, and Duffy blood groups [4]. ABO and Rh typing results in a chance of compatible transfusion 99.8% of the time. The addition of cross-matching (testing the recipient serum with donor red blood cells (RBCs)) and antibody testing (donor serum tested with commercially prepared antigen) increases donor-recipient compatibility to 99.95%. However, even with identification of a rare blood group on transfusion testing, a treatment conundrum still exists if there are no donor blood products compatible with these rare blood groups. The following case presentation will review such considerations of a rare blood group to include pre-operative and post-operative management.

## Case presentation

A 37-year-old gravida 2 para 1 with one prior full-term singleton pregnancy with a past medical history of chronic hypertension and multiple uterine fibroids at 39 weeks and five days estimated gestational age (EGA) presented for scheduled elective repeat CS as well as a bilateral tubal ligation for contraception. The patient's prior pregnancy history was notable for an uncomplicated elective CS secondary to her history of intramural fibroids. Her current prenatal course was notable for a positive antibody screen in G1 and in the current pregnancy that was closely followed by both hematology and the blood bank for Junior blood group system negative blood (Jr(a-)). Her diagnosis was confirmed with anti-Jr(a) antibodies detected by monolayer assay (MMA), the current gold standard technique for both detecting and assessing the clinical significance of irregular antibodies. Her preoperative laboratory values were notable for hemoglobin (Hgb) of 12.3 g/dL, hematocrit (Hct) of 37.5%, and platelets (PLT) of 161 × 10^3^/µL.

Following both consultation with hematology and the blood bank, it was determined that the patient's presence of anti-Jr(a) antibodies was “extremely clinically significant” and that the patient's blood was incompatible with 99.9% of available blood products in the United States of America. The anesthesiology team contacted the blood bank for clarification on the pertinent Jr(a-) antibody results and any recommended contingency planning if the patient required blood product resuscitation. The conclusion of this multidisciplinary discussion was that there are no available blood products she could receive without significant risk of hemolysis, and in the event of last line therapy, she would have to receive incompatible products. Via multidisciplinary discussion, the team elected that the most reliable contingency in the event of perioperative hemorrhage would be utilization of a cell saver. Given that the patient was within three days of CS, autologous blood donation was not considered at the time of presentation.

None of the team members caring for this high-risk obstetric surgical patient had ever encountered a complex clinical dilemma of proceeding with a CS with an elevated risk of hemorrhage without the ability to safely transfuse matched RBCs in the event of an emergency or hemodynamic instability. This hematologic challenge altered both anesthetic and surgical planning. The original anesthetic plan was for a single-shot spinal (intrathecal) anesthetic with 1.6 mL of 0.75% bupivacaine, 100 µg epinephrine, 100 µg morphine, and 15 µg of fentanyl. Following a discussion with the obstetric surgeons who felt they could significantly reduce potential hemorrhage by employing a slower, more deliberate, and methodical surgical approach, the anesthesia team elected for a combined spinal epidural (CSE) to provide adequate analgesia for the duration of the surgery. 

Upon agreement of both the anesthetic and surgical plan, the patient was brought to the operating room (OR) and connected to standard American Society of Anesthesiologists (ASA) monitors with supplemental oxygen administered via nasal cannula at 4 L per minute. A 500 mL bolus of lactated ringers (LR) was given prior to neuraxial anesthesia. At this time, a CSE was performed without complication with the identical single-shot dosages previously aforementioned. Of note, the patient did not exhibit any hypotension, nausea, or fetal bradycardia associated with spinal anesthetic. A spinal level was then checked and determined to provide adequate analgesia to T4 dermatomes bilaterally. To further mitigate risk of hemorrhage, 1,000 mg of tranexamic acid (TXA) was administered during skin incision and an additional second-line appropriate uterotonic in 250 µg of carboprost tromethamine was also prepared. The patient's history of chronic hypertension portended methergine contraindication.

The initial, methodical surgical dissection down to the uterus proceeded without complication, however, upon uterine incision, brisk bleeding was encountered secondary to known fibroid disease. The fetus was delivered without complication and per hospital protocol three-unit oxytocin boluses were administered every three minutes for a total of three boluses (nine units total administered over nine minutes). Following the third bolus, the oxytocin was placed on a maintenance rate of 95 mL/hour (six units an hour). Despite rapid administration of oxytocin, the patient continued to have intrauterine blood loss from the hysterotomy incision while uterine tone remained poor; 5% albumin was administered as a temporizing measure for volume expansion. Red cell salvage from intraoperative bleeding was obtained via cell saver with a plan to auto transfuse once hemostasis was achieved. Carboprost tromethamine (250 µg) was given intramuscularly at this time to address residual uterine atony. During this part of the case, the patient transiently required a phenylephrine infusion between 40 and 75 µg/minute to ensure her mean arterial pressure was maintained above 65 mmHg. Surgical control of the bleeding was achieved with surgical measures, the hysterotomy incision was closed, and the scavenged blood via cell saver was packaged and prepared with a total of 387 mL collected. The blood was immediately transfused via fluid warmer without adverse reaction, and the patient's vasopressor requirement was quickly titrated off. After surgical closure, she was transferred hemodynamically stable without pain to the post-anesthesia recovery unit for a total intraoperative time of 2.5 hours. 

## Discussion

The International Society of Blood Transfusions Working Party for Red Cell Immunogenics and Blood Group Terminology is a collective of international experts that develop and maintain guidelines for hematological scientific research to include blood group antigen and allele nomenclature. As of 2024, there are currently 45 individual blood group systems containing 362 unique red cell antigens. The term “blood group” refers to the entire blood group system comprising RBC antigens whose unique specificity is controlled upstream by a series of genes on the same chromosome [4]. The variation in blood group systems is genetically determined by approximately 50 genes [5]. Of the blood groups, the ABO and Rh blood group systems are among the most polymorphic, immunogenic, and clinically important in humans for transfusion and transplantation [6]. However, many other blood group systems beyond ABO and Rh can be clinically significant, including the Jr(a) negative blood group.

**Figure 1:**
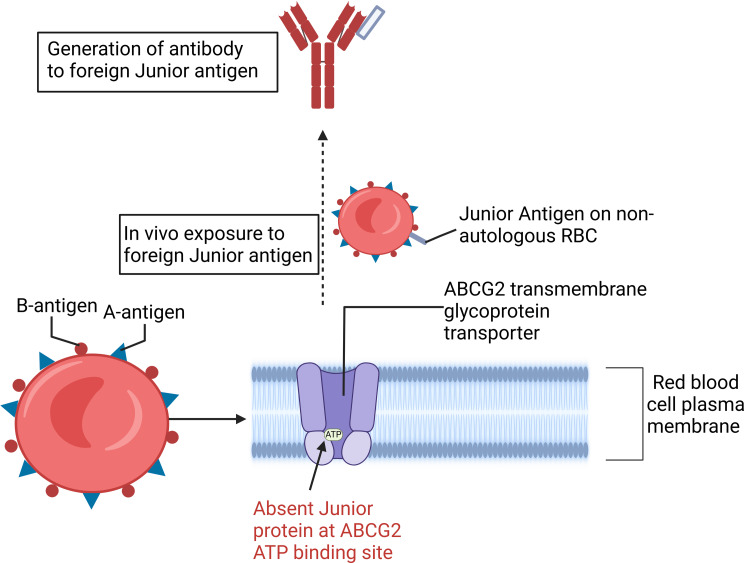
IgG antibody production against Junior antigen located on ABCG2 transmembrane glycoprotein Figure created by the authors using BioRender.

This Jr(a) negative blood group phenotype most commonly results from recessive inheritance of ABCG2 null alleles caused by a frameshift or nonsense mutation [7]. In almost all cases, the initial mutation introduces a premature stop codon in the ABCG2 gene on chromosome 4, which encodes an ATP-binding site of the ABCG2 transporter on erythrocytes [7,8]. With this mutation, the patient’s erythrocytes will lack the Jr(a) antigen on the membrane glycoprotein surface of the ABCG2 and are therefore susceptible to IgG antibody production against the Jr(a) antigen in the event of antigen exposure (Figure 1) [8,9]. This antibody is not thought to be naturally occurring, and its production is most commonly stimulated by pregnancy or transfusion [10].

The process of identifying and diagnosing rare blood groups such as the Jr(a) patient will largely depend on clinical context. For the typical obstetric surgical patient, a preoperative decision is made as to the level of pretransfusion testing required for the individual patient and surgical procedure. Typically, this is either no pretransfusion testing, type and screen, or type and cross with a requested number of units to be prepared prior to an operation. This decision is generally based on physician acumen and judgment, prior known diagnoses, the patient's preoperative Hgb/hematocrit concentration, and anticipated surgical blood loss [11]. Institutional pretransfusion processes are in place to avoid any potential blood matching errors and mitigate potentially fatal complications, such as hemolytic transfusion reactions typically caused by ABO incompatibility, leading to acute complement-mediated intravascular hemolysis. In the event of pre-operative blood testing, the Jr(a) negative phenotype may be diagnosed after a positive indirect Coombs test that necessitates further antibody testing. Antibody reactions in the anti-Jr(a) population are then confirmed by a monolayer antibody assay, as is the case in this patient. In the event of a diagnosis of a Jr(a) negative blood group, clinical context will determine the next steps for operative or non-operative planning.

For the vast majority of patients, the transfusion process is completed and carried out without issue or complication. For example, of the eight common ARO Rh blood types, AB negative (AB-) is only present in an estimated 0.67% of all Americans (one out of 149 individuals). Despite its incredibly low prevalence in the population, there is very little demand or shortages of AB- blood. AB- individuals enjoy the added benefit of being compatible with any Rh negative donor, which is approximately 7% of the population. The dilemma presents itself (as in this case) when managing a patient with a significantly rare blood type that is not compatible with the eight common blood types. Although the hospital multidisciplinary team followed the appropriate pretransfusion testing algorithm, the patient’s blood reacting with all RBCs in the antiglobulin phase of testing, with suspected single red cell antibody directed at a high frequency antigen, made blood transfusion intensely problematic. Fortunately, the patient was not in a situation requiring emergency blood and had the benefit of having multiple appointments and diagnostic tests performed with the blood bank to correctly elucidate the Jr(a) negative blood type. Despite previous knowledge of the condition, the patient was told that “there are no available blood products that she could receive in the country” and that should she require transfusion in an emergency, she would “have to receive incompatible blood" given that no prior autologous transfusion was performed. In addition to maternal risks, incompatible blood would be especially problematic prior to delivery as the Jr antigen is most strongly expressed on cord blood cells which places the neonate at elevated risk of hemolytic disease. 

This was a complex and challenging circumstance for both the OR team and the patient and significantly altered how the surgery and anesthetic were performed. Preoperatively, close coordination with hematology and the blood bank service was essential for identifying and sourcing any potentially available matched blood products. Intra-operatively, a slow, methodical dissection provided optimal hemostatic control. From an anesthetic approach, the CSE offered flexibility and the ability to re-dose epidural medications in a slow, controlled manner, which can further optimize surgical conditions. TXA was used to help minimize surgical bleeding in this high risk patient. Albumin was used for quick volume expansion, and, lastly, the implementation of blood cell salvaging techniques, such as the cell saver, was particularly advantageous for resuscitation given that no compatible blood products are available. 

In addition to the above management strategies, clinicians can consider administering erythropoietin with supplemental iron two to three weeks prior to surgery. Finally, autologous blood donation is a less practiced but potential option of collecting the patient's blood in advance to have it prepared and ready for administration during surgery. However, fetal monitoring, more frequent doctors’ appointments, advanced planning, and risks of donating blood may make autologous blood donation burdensome to some patients. 

## Conclusions

Although it is among the rarest of all clinically significant blood types, peripartum patients with Jr(a) negative blood groups who develop anti-Jr(a) antibodies are at significantly elevated perioperative risk due to hemorrhage, transfusion reactions, and difficulty in obtaining properly matched blood products for safe transfusion. The aforementioned case presented a 37-year-old gravida 2 para 1 with one prior full-term singleton birth presenting for a scheduled CS who was identified to have the Jr (a) negative blood group. Through meticulous multidisciplinary planning and perioperative vigilance, the patient ultimately had a successful CS with a viable term infant without the need for incompatible blood product transfusion. It is the recommendation of the authors that clinicians carry a heightened awareness for rare blood groups in the obstetric setting and are prepared to mitigate risk in this medically challenging population.
